# P‑Type Spin-Coated Sol–Gel Cr_2_O_3_ Layer by Postannealing in Vacuum

**DOI:** 10.1021/acsomega.5c02540

**Published:** 2025-08-05

**Authors:** Wei-Chih Lai, Chan-Hung Hsu, Bo-Ting Wu, Po-Han Chen, Sheng-Po Chang, Cheng-Huang Kuo, Jinn-Kong Sheu, Shoou-Jinn Chang

**Affiliations:** † Department of Photonics, 34912 National Cheng Kung University, Tainan 70101, Taiwan; ‡ Research Center for Energy Technology and Strategy, National Cheng Kung University, Tainan 704, Taiwan; § Program on Key Materials, Academy of Innovative Semiconductor and Sustainable Manufacturing, National Cheng Kung University, Tainan 70101, Taiwan; ∥ Master Degree on Nano-integrated Circuit Engineering and Department of Electrical Engineering, 593397 National Cheng Kung University, Tainan 701, Taiwan; ⊥ Department of Microelectronics Engineering, 517768 National Kaohsiung University of Science and Technology, Kaohsiung 81157, Taiwan; # Institute of Lighting and Energy Photonics, College of Photonics, National Yang Ming Chiao Tung University, Tainan 71150, Taiwan; ∇ Department of Electrical Engineering, 34912 National Cheng Kung University, Tainan 70101, Taiwan

## Abstract

The hole carriers of spin-coated sol–gel Cr_2_O_3_ could be activated successfully by postannealing at a temperature of more than 500 °C in a vacuum without doping. The sheet hole concentration increased with the increase in the postannealing temperature and could reach 5 × 10^14^ cm^–2^ at a postannealing temperature of 680 °C. However, activating the hole carriers for annealing Cr_2_O_3_ in N_2_ ambient at a low pressure of 3 mTorr and a temperature of 560 °C or higher was difficult. The N_2_-annealed Cr_2_O_3_ could regain the same electrical properties as vacuum-annealed Cr_2_O_3_ by annealing under vacuum again. A comparison of the deconvoluted XPS spectra of the postannealed Cr_2_O_3_ in a vacuum and in N_2_ ambient showed that the amount of Cr^2+^, Cr^4+^, Cr^5+^, Cr^6+^, and O vacancies was strongly correlated with the hole carrier creation of Cr_2_O_3_.

## Graphical abstract

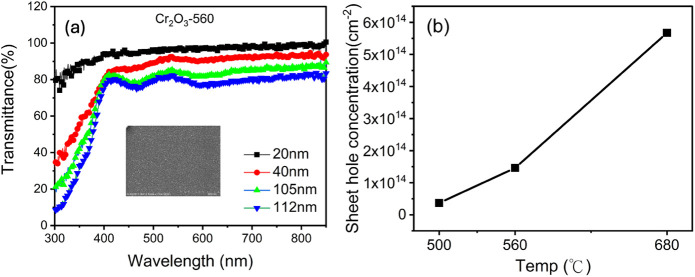

## Introduction

Transparent conducting oxides (TCOs) have high optical transparency and electrical conductivity. This characteristic comes from the requirements of having the first allowed optical transition at energy values higher than 3.1 eV and having uncompensated shallow donor (or acceptor) states close to the bottom (top) of the conduction band minimum (valence band maximum).^1–4^ Most TCOs are n-type^5,6^ and could be applied to a broad range of applications in optoelectronic devices such as light-emitting diodes, flat panel displays, thin-film solar cells, touch screens, and liquid crystal displays.^7,8,9^ The p-type TCOs are crucial for realizing fully transparent CMOS-type electronics.^10^ Moreover, p-type TCOs performed well in hole-injecting or extracting layers to improve the efficiencies of organic light-emitting diodes or photovoltaic solar cells.^11–14^ However, p-type TCOs displayed optoelectrical characteristics that were poorer than those of their n-type counterparts. The low conductivity in most p-type TCOs is due to oxygen p-states dominating the top of the valence band of p-type TCOs, which are highly localized and lead to flat bands and, consequently, a large effective hole mass.^15^ Kawazoe et al.^16^ reported delafossite-structured CuAlO_2_ p-type TCOs with a conductivity of 1 S/cm and an optical band gap of 3.1 eV. They introduced a trivalent cation, Al, to delocalize the valence band edge because the trivalent cation outer shell orbital hybridized with the anion 2p orbital. The Mg-doped CuCrO_2_ thin film would reach a p-type conductivity of 220 S/cm, but its transparency was around 30%.^17^ However, the dopant distribution and dephasing of the ternary oxide would cause large variations in the electrical properties of this doped ternary oxide and challenge the deposition conditions.^18–21^

Chromium oxide (Cr_2_O_3_) has recently received increasing interest as a p-type TCO due to its wide optical band gap of 3.1 eV.^22^ In contrast to the doped ternary oxide, Cr_2_O_3_ is a thermodynamically most stable TCO that would avoid the effects of dopant distribution and dephasing. However, it has low conductivity, which needs to be improved by using dopants such as Mg, Zn, Ni, Cu, In, and co-dopants like Mg and N.^23–34^ The p-type Cr_2_O_3_ without doping could be prepared under high-temperature oxidation at low partial O_2_ pressure.^35^ The Cr_2_O_3_ usually has remarkably high resistance and has a very low reported native conductivity. Therefore, obtaining p-type Cr_2_O_3_ without doping is a challenge.^32,36–38^ In this report, we demonstrate the synthesis of p-type Cr_2_O_3_ without doping using easy, moderate-temperature, and time-saving methods. The solution-based deposition method is simple, is free of a vacuum system, and is low-cost for starting precursors compared to the vacuum-based deposition methods. The optoelectrical properties of Cr_2_O_3_ without doping are investigated with respect to its preparation conditions.

## Experimental Section

An almost 20-nm-thick Cr_2_O_3_ was deposited on a (0001) sapphire substrate by applying the spin coating process with a sol–gel precursor. Before deposition of the Cr_2_O_3_ layer, we prepared a Cr_2_O_3_ sol–gel solution by dissolving 1.6 mmol of chromium­(III) nitrate nonahydrate (Cr­(NO_3_)_2_•9H_2_O) in 1 mL of 2-methoxyethanol, 1 mL of acetic acid, and 2 mL of polyethylene glycol. We then stirred the prepared Cr_2_O_3_ solution at a temperature of 400 °C for more than 12 h. The Cr_2_O_3_ sol–gel solution was spun-cast at 6000 rpm for 60 s. The spin-coated Cr_2_O_3_ sol–gel on a sapphire substrate was then baked at 400 °C on a hot plate in the atmosphere for 15 min to form an as-deposited Cr_2_O_3_ layer. The as-deposited Cr_2_O_3_ layers were postannealed in a quartz furnace at various temperatures and ambient conditions for 1.5 h. The temperatures for the postannealing were 450 °C, 560 °C, and 680 °C. The postannealing ambient conditions were in a vacuum with a background pressure of 3 mTorr and N_2_ 5 sccm with a pressure of 45 mTorr. The Cr_2_O_3_ sample was denoted as Cr_2_O_3_-temperature-V; for example, Cr_2_O_3_-450-V represents the Cr_2_O_3_ post-annealed at that temperature in a vacuum ambient. Cr_2_O_3_-temperature-N_2_, for instance, Cr_2_O_3_-560-N_2_, would refer to Cr_2_O_3_ postannealing at that temperature in N_2_ ambient. The Cr_2_O_3_-temperature-N_2_–V—for instance, Cr_2_O_3_-560-N_2_–V—would represent the Cr_2_O_3_ postannealing at that temperature in N_2_ ambient and annealing in a vacuum again.

The material and optical properties of the prepared Cr_2_O_3_ layers with different postannealing temperatures and ambient conditions, such as their morphology, material quality, and transmittance, were characterized via scanning electron microscopy (SEM, SU8000), X-ray diffraction (XRD, Bruker), grazing-incidence wide-angle X-ray scattering, X-ray photoelectron spectroscopy (XPS, PHI VersaProbe 4), transmittance, and absorbance (UV/vis, U-4100) measurements. The electrical properties of all Cr_2_O_3_ samples, such as resistivities (or conductance) and carrier concentrations, were measured by a four-point probe (LRS4-T) and Hall measurement (HL990).

## Results and Discussion

Figure 1 shows the top-view and cross-sectional SEM images of the as-deposited Cr_2_O_3_ and postannealed Cr_2_O_3_ at different temperatures in a vacuum. The as-deposited Cr_2_O_3_ and the Cr_2_O_3_ post-annealed at different temperatures had a thickness of around 10 to 15 nm from their cross-sectional images. The as-deposited Cr_2_O_3_ presents a featureless surface in the top-view SEM image in Figure 1. The grains appeared on the surface after as-deposited Cr_2_O_3_ was annealed at different temperatures in a vacuum. The grain size increased with the increase in the annealing temperature. The largest grain size of 13.9 nm was achieved at an annealing temperature of 680 °C. The as-deposited Cr_2_O_3_ might aggregate or recrystallize during annealing at various temperatures in a vacuum. This phenomenon turned the featureless surface of the as-deposited Cr_2_O_3_ into a small-grain-featured surface after annealing in a vacuum.

**Figure 1:**
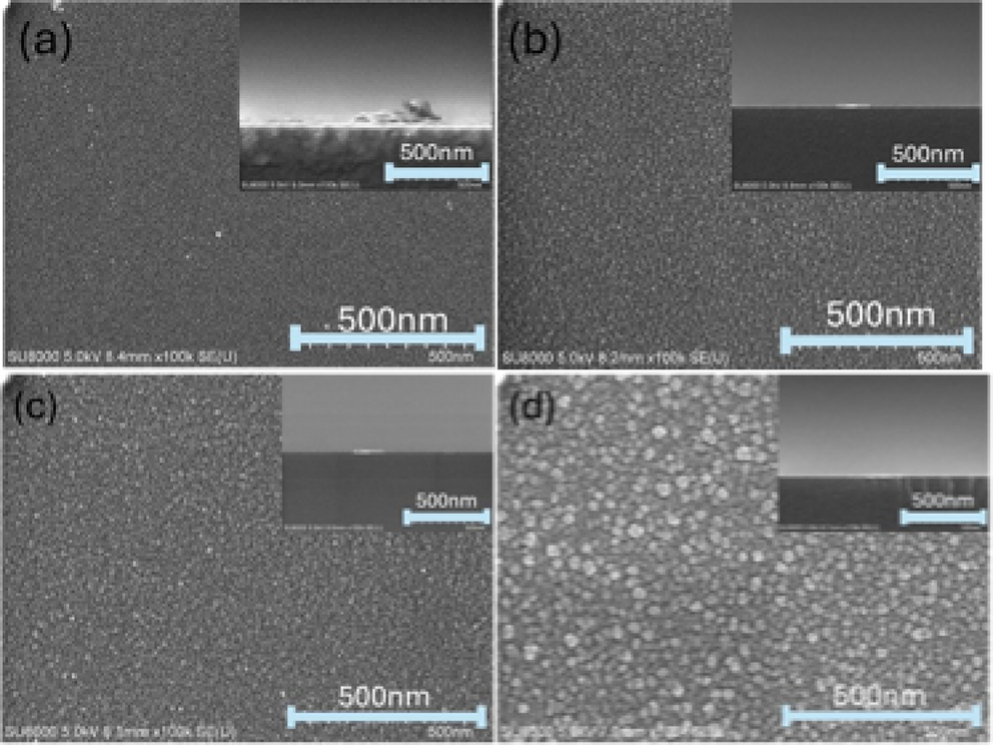
SEM images of the as-deposited Cr_2_O_3_ and postannealed Cr_2_O_3_ at different temperatures in a vacuum: (a) 450 °C, (b) 500 °C, (c) 560 °C, and (d) 680 °C.

Figure 2a shows the transmittance of the 20-nm-thick Cr_2_O_3_ with and without annealing in a vacuum and in N_2_ ambient. The as-deposited Cr_2_O_3_ films present an almost constant transmittance of more than 90% from the wavelength of 850 nm to nearly 400 nm. The transmittance of the as-deposited Cr_2_O_3_ slowly decreases to 80% in the wavelength range from 400 to 300 nm. In other words, the as-deposited Cr_2_O_3_ presented.

**Figure 2:**
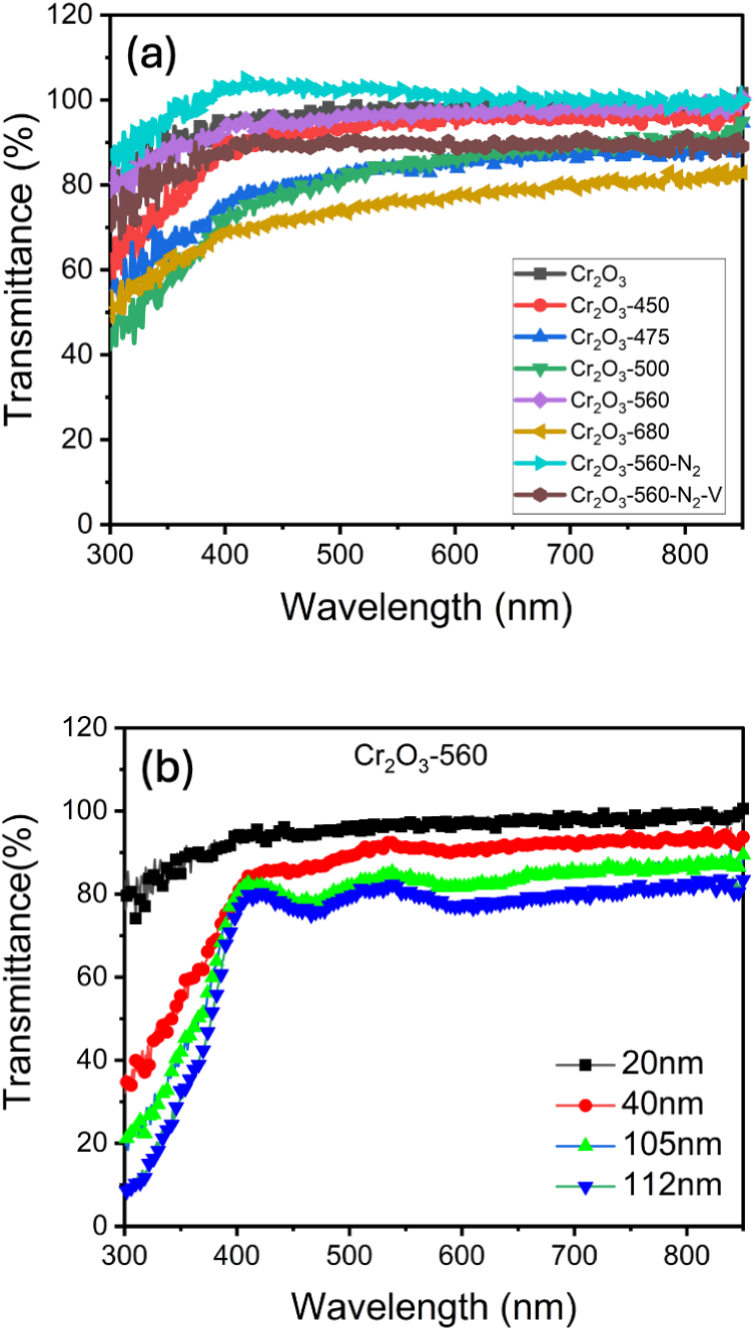
(a) Transmittance spectra of the 20-nm-thick Cr_2_O_3_ with and without annealing in a vacuum and in N_2_ ambient (b) The transmittance spectra of the 560 °C-annealed Cr_2_O_3_ layer with thicknesses of 20, 40, 105, and 112 nm.

light absorption for wavelengths less than 400 nm, the band gap of the Cr_2_O_3_. The transmittance of the postannealed Cr_2_O_3_ decreased with the increase in the annealing temperature. The transmittance of 450 °C annealed Cr_2_O_3_ was slightly less than that of as-deposited Cr_2_O_3_ in the 400–800 nm wavelength range. Similar to as-deposited Cr_2_O_3_, the transmittances of 450-°C annealed Cr_2_O_3_ dropped for wavelengths less than 400 nm. CrO annealed at 475 °C, 500 °C, and 680 °C presented a large decrease in transmittance compared to as-deposited Cr_2_O_3_ in the wavelength range of 400–800 nm. Moreover, the transmittance of 475 °C, 500 °C, and 680 °C annealed Cr_2_O_3_ showed a gradual decrease with decreasing wavelength from 400 to 800 nm range and declined quickly for wavelengths less than 400 nm. However, the 560 °C annealed Cr_2_O_3_ layer presented a larger transmittance than that of the 475 °C, 500 °C, and 680 °C annealed Cr_2_O_3_ layers. After annealing, the reduction in transmittance for wavelengths larger than 400 nm in post-annealed Cr_2_O_3_ might be attributed to enhanced light scattering. From the observation of the top-view SEM images of postannealed Cr_2_O_3_ in Figure 1, the increase in the annealing temperatures enlarged the grain features of Cr_2_O_3_, thereby enhancing light scattering for wavelengths less than 400 nm. This phenomenon results in the lower transmittance of postannealed Cr_2_O_3_ than that of as-deposited Cr_2_O_3_. However, the high-temperature postannealing might enhance the defect absorption, resulting in a gradual decrease in transmittance in the wavelength range from 400 to 800 nm.

Figure 2b shows the transmittance spectra of the 560 °C-annealed Cr_2_O_3_ layer with different thicknesses. The transmittance in the visible wavelength range (400–850 nm) reduces as the thickness of the Cr_2_O_3_ layer increases. The transmittance of the Cr_2_O_3_ layer at a wavelength of 550 nm would drop roughly from 92% to 81% when the thickness increases from 20 to 110 nm. The average transmittance of a 110-nm-thick Cr_2_O_3_ layer postannealed at 560 °C could still be nearly 80%.

The postannealing temperature significantly influenced the electrical properties of Cr_2_O_3_. Figure 3a shows the sheet resistance of Cr_2_O_3_ at various annealing temperatures under vacuum by using a four-point probe. The as-deposited Cr_2_O_3_ had very high resistance, far beyond the four-point probe’s measurement limitation. The 450 °C-annealed Cr_2_O_3_ presented a large resistance that was outside the equipment’s limits. Therefore, showing the sheet resistance of as-deposited and 450 °C-annealed Cr_2_O_3_ in Figure 3a is impossible. The sheet resistances of postannealed Cr_2_O_3_ declined with the increase in the annealing temperature. The sheet resistance showed a fast decrease in the annealing temperature range of 475–560 °C and was almost saturated for the annealing temperature more than 560 °C. The 680 °C annealed Cr_2_O_3_ presented the lowest sheet resistance of 150 kΩ. The reduction of the sheet resistance of postannealed Cr_2_O_3_ should be attributed to the successful activation of the carriers in Cr_2_O_3_ by thermal annealing in a vacuum. The Hall measurement determined the carrier type and concentration of postannealed Cr_2_O_3_, revealing that the carrier type was a hole. Figure 3b shows the hole concentrations at various annealing temperatures. The sheet hole concentrations of Cr_2_O_3_ rose with the increase in the annealing temperature. The hole concentration could reach the highest value of 5 × 10^14^ cm^–2^ for Cr_2_O_3_ annealed at 680 °C. The Hall measurement with various temperatures was performed on the 500 °C, 560 °C, and 680 °C annealed Cr_2_O_3_ to obtain the temperature-dependent hole concentrations. The temperature-dependent hole concentrations of 500 °C, 560 °C, and 680 °C annealed Cr_2_O_3_ are shown in Figure S1. The hole activation energies of 500 °C, 560 °C, and 680 °C annealed Cr_2_O_3_ could be found in the Arrhenius plot of temperature-dependent hole concentrations. Figure 3c presents the hole activation energies with the annealing temperatures. The hole activation energies decreased with an increase in the annealing temperatures. The low hole activation energy resulted in a high hole concentration at room temperature. Therefore, the 680 °C-annealed Cr_2_O_3_ has the highest sheet hole concentration and the lowest sheet resistance because of the lowest hole activation energy of 0.16 eV.

**Figure 3:**
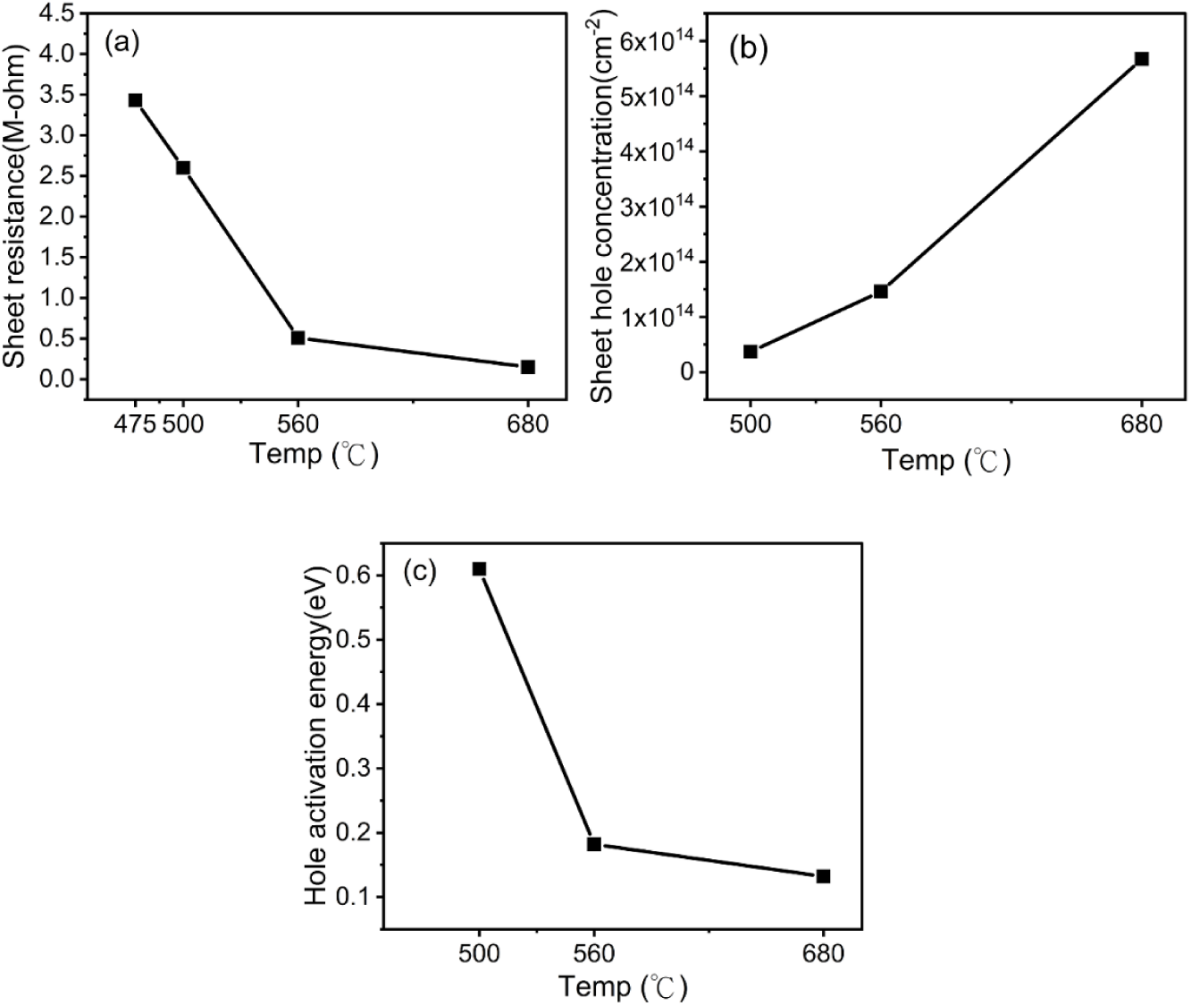
(a) Sheet resistance of Cr_2_O_3_ with various annealing temperatures in a vacuum measured using a four-point probe. (b) Hole concentrations of Cr_2_O_3_ with various annealing temperatures. (c) Hole activation energies of Cr_2_O_3_ with the annealing temperatures.

XPS was applied to characterize the components of the postannealed Cr_2_O_3_ layers with various annealing temperatures to understand the material’s chemistry upon postannealing in a vacuum. The XPS core-level spectra of the O 1s and Cr 2p of Cr_2_O_3_ layers with various annealing temperatures in a vacuum are shown in Figure 4. The XPS spectra of O and Cr were performed and deconvoluted using XPSPEAK41 software, which determines the coherent fitting of peaks to the core level. Figure 4a presents the core-level spectra of Cr 2p of Cr_2_O_3_ at various annealing temperatures. The Cr_2_O_3_ with different annealing temperatures showed similar core-level spectra of Cr 2p with two main peaks around 576.5 and 586.3 eV, which are assigned to Cr 2p_3/2_ and Cr 2p_1/2_, respectively.^39^ The core-level spectra of Cr 2p_3/2_ of Cr_2_O_3_ could be deconvoluted into the oxidation states of Cr^2+^, Cr^3+^, Cr^4+^, Cr^5+^, and Cr^6+^ with peak positions of 575.42, 576.46, 577.34, 578.32, and 579.22 eV, respectively.^40^ The annealing temperature-dependent amounts of Cr in different oxidation states in Cr_2_O_3_ are shown in Figure 4b. It was found that the annealing temperatures of Cr_2_O_3_ modified the amounts of all the Cr oxidation states in Cr_2_O_3_. The amount of Cr with high oxidation states showed an increase in the annealing temperature, while the amounts of Cr^2+^ and Cr^3+^ caused a decrease in the annealing temperature. Cr^2+^ had a steeper decline in amount than Cr^3+^.

**Figure 4:**
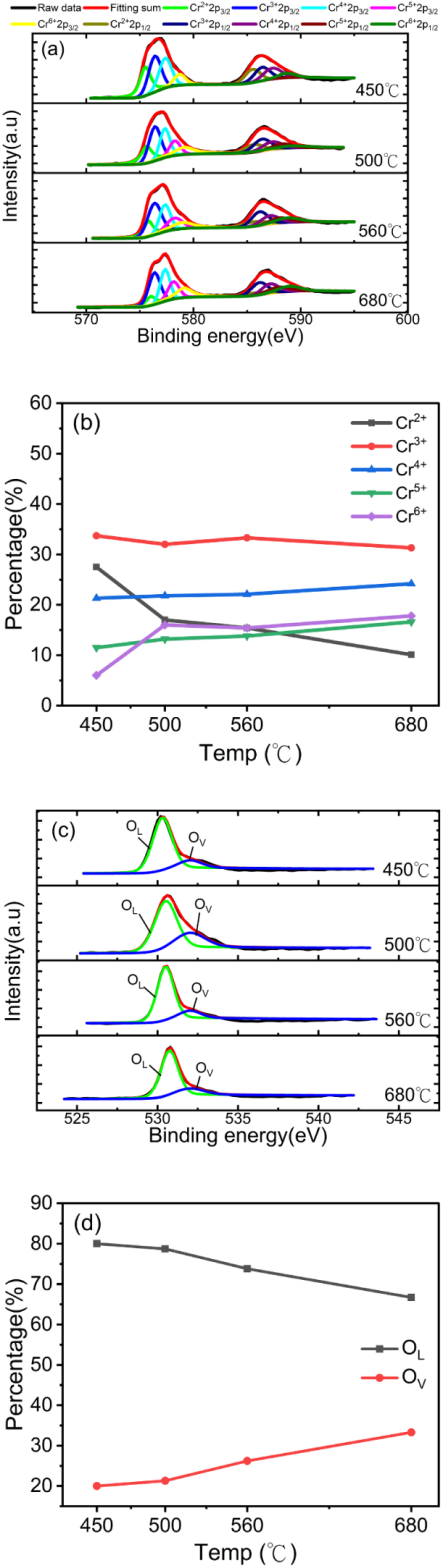
(a) Core-level spectra of Cr 2p of Cr_2_O_3_ with spectra deconvolution at various annealing temperatures. (b) The amount of Cr^2+^, Cr^3+^, Cr^4+^, Cr^5+^, and Cr^6+^ with the annealing temperatures. (c) The annealing temperatures modified the O 1s core-level spectra of Cr_2_O_3_ with spectral deconvolution. (d) The quantities of the O_L_ and O_V_ increased with the annealing temperatures of Cr_2_O_3_.

The annealing temperatures modified the core-level O 1s spectra of Cr_2_O_3_ in Figure 4c. The main peak binding energy of the O 1s core-level spectra of Cr_2_O_3_ drifted toward the high binding energy with increased annealing temperatures. The core-level spectra of O 1s of Cr_2_O_3_ with different annealing temperatures were then deconvoluted into the lattice oxygen (O_L_) and vacancy oxygen (O_V_) of Cr_2_O_3_ spectra at peak binding energies of 529.98 and 532.71 eV, respectively, to understand the detailed changes in O’s chemical binding with respect to the annealing temperatures.^41,42^ The quantities of the O_V_ increased with the increase in the annealing temperatures of Cr_2_O_3_, as shown in Figure 4d. The amounts of Cr^2+^, Cr^4+^, Cr^5+^, Cr^6+^, and O_v_ might play a crucial role in the hole concentration of Cr_2_O_3_ from the XPS deconvolution results of all Cr oxidation states and O_v_ at different annealing temperatures.

The electrical property results and XPS deconvolution results of Cr_2_O_3_ with different annealing temperatures in this study seem to conflict with those of previous reports. Many studies in experimental research^43–55^ and theoretical calculations^56,57^ have reported that the defect types in Cr_2_O_3_ influence its electrical properties, including the carrier type. Prior studies have shown that the Cr vacancy is the dominant parameter for hole carrier formation in Cr_2_O_3_. Moreover, annealing at high O_2_ partial pressure is advantageous in forming the Cr vacancies in the Cr_2_O_3_. The vacancies of O_2_ in Cr_2_O_3_ would prefer the formation of electrons in Cr_2_O_3_. However, in this study, p-Cr_2_O_3_ could be produced at an annealing temperature of more than 500 °C in a vacuum. Moreover, the postannealed p-Cr_2_O_3_ with detectable hole concentration showed increased O vacancies. According to the conclusions of previous reports, annealing in a vacuum and high O vacancies should be favorable for forming electrons but for forming holes in Cr_2_O_3_. However, the Cr_2_O_3_ layer showed increased amounts of Cr^6+^ for the postannealing temperatures higher than 500 °C in Figure 4b. The presence of Cr^6+^ implied the compound CrO_3_, which has a low boiling point of 250 °C, in the Cr_2_O_3_. The compound of CrO_3_ in Cr_2_O_3_ may enhance the creation of the Cr and O vacancies during postannealing at high temperatures in a vacuum because of the evaporation of the CrO_3_ component. In Figure 4b,c, the Cr_2_O_3_ presented increased amounts of Cr^6+^ and O vacancy defects for the Cr_2_O_3_ annealed temperature higher than 500 °C. In other words, the Cr_2_O_3_ with an annealing temperature of more than 500 °C would have higher quantities of Cr vacancies than the Cr_2_O_3_ with an annealing temperature of less than 500 °C. Therefore, the Cr_2_O_3_ with an annealing temperature of more than 500 °C in a vacuum could present a high hole concentration.

In addition, the amount of Cr^4+^ would also affect the hole concentration of Cr_2_O_3_. Cojocaru^54,55^ reported that a small amount of unoccupied Cr^4+^ states in Cr_2_O_3_ hopped from one lattice chromium to another at temperatures above 350 °C. This phenomenon resulted in the p-type conductivity of Cr_2_O_3_. The results of XPS deconvolution showed that the composition of Cr^4+^ increased with an increase in annealing temperature. The increased composition of Cr^4+^ would increase the hole concentration of Cr_2_O_3_, resulting in the largest sheet hole concentration of 680 °C annealed Cr_2_O_3_.

It was also found that the amount of Cr^2+^ rapidly declined with the increase in the annealing temperature because Cr_2_O_3_ has a high hole concentration at high annealing temperatures. The amount of Cr^2+^ should have an effect on the hole concentration of Cr_2_O_3_. The theoretical simulations reported that Cr^2+^ interstitial in Cr_2_O_3_ would have a very deep transition level or serve as donors for electron carriers.^56,57^ The high amounts of Cr^2+^ might suppress the hole concentration of Cr_2_O_3_.

Activating the hole carriers for annealing Cr_2_O_3_ in ambient N_2_ at a low pressure of 45 mTorr and a temperature of 560 °C or even higher was difficult. However, the hole carriers of N_2_-annealed Cr_2_O_3_ could be reactivated when N_2_-annealed Cr_2_O_3_ was annealed in a vacuum again. The electrical properties of the N_2_-annealed Cr_2_O_3_, such as the hole carrier concentrations and resistance shown in Table 1, were the same as those of Cr_2_O_3_ annealed in a vacuum at the same annealing temperature. This phenomenon seems to be reversible. In the previous discussion, the amounts of Cr^2+^, Cr^4+^, Cr^5+^, Cr^6+^, and O_v_ played a crucial role in creating the hole carrier in Cr_2_O_3_. Therefore, the XPS core-level spectra with spectral deconvolution of Cr 2p and O 1s in Figure 5a,c were compared for Cr_2_O_3_ annealed at 560 °C in a vacuum, in N_2_ ambient, and for N_2_-annealed Cr_2_O_3_ annealed in a vacuum to understand the material’s chemical differences in these three samples.

**Figure 5:**
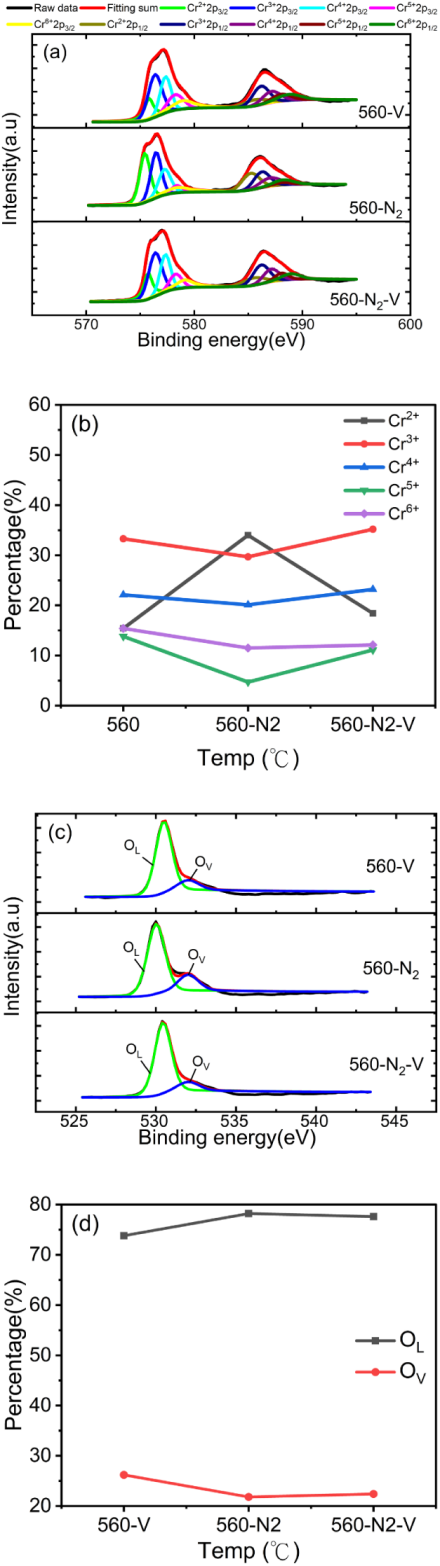
XPS core-level spectra with spectral deconvolution of (a) Cr 2p and (c) O 1s for Cr_2_O_3_ annealed at 560 °C in a vacuum, in N_2_ ambient, and the N_2_-annealed Cr_2_O_3_ subsequently annealed in a vacuum. (b) The amount of Cr^2+^, Cr^3+^, Cr^4+^, Cr^5+^, and Cr^6+^, and (d) the amounts of O_L_ and O_V_ with Cr_2_O_3_ annealed at 560 °C in a vacuum, in N_2_ ambient, and the N_2_-annealed Cr_2_O_3_ subsequently annealed in a vacuum.

**Table 1:** Summary of the Measured Electrical Properties of Cr_2_O_3_ Annealed at 560 °C in a Vacuum and in N_2_ Ambient, and the Subsequent Annealing of N_2_-annealed Cr_2_O_3_ in a Vacuum

	Sheet carrier concentration (cm^–2^)	Resistance (M-ohm)	Work function (meV)	Hole activation energy(eV)
560-Vac	$1.459\times 10^{14}$	0.51	5.0983	0.182
560-N_2_	NA	NA	4.8342	NA
560-N_2_–Vac	$2.102\times 10^{14}$	0.43	5.0102	0.172

Figure 5b,d show the amounts of Cr^2+^, Cr^3+^, Cr^4+^, Cr^5+^, Cr^6+^, and O_V_ extracted from the deconvolution of Cr 2p and O 1s for Cr_2_O_3_ annealed at 560 °C in a vacuum and in ambient N_2_ and the N_2_-annealed Cr_2_O_3_ annealed again in a vacuum. The Cr_2_O_3_ annealed in ambient N_2_ presented the lowest Cr^4+^, Cr^5+^, Cr^6+^, and O_V_ amounts and the largest Cr^2+^ amount. The Cr_2_O_3_ annealed in N_2_ might suppress the formation of Cr^4+^, Cr^5+^, Cr^6+^, and the amount of O_v_, while keeping Cr^2+^ at a high level. Therefore, the Cr_2_O_3_ annealed in ambient N_2_ presented high resistance and an undetectable carrier type and concentration. The amounts of Cr^4+^, Cr^5+^, Cr^6+^, and O_v_ rose to levels similar to those of Cr_2_O_3_ annealed again in a vacuum when N_2_-annealed Cr_2_O_3_ was subjected to vacuum annealing. In addition, the Cr^2+^ amount could be reduced to a low level when N_2_-annealed Cr_2_O_3_ was annealed again in a vacuum. The N_2_-annealed Cr_2_O_3_ that was annealed again in a vacuum could thus have a high hole sheet concentration of 2.1 × 10^14^ cm^–2^, which is almost the same as that of the Cr_2_O_3_ annealed in a vacuum. Therefore, the spin-coated sol–gel Cr_2_O_3_ annealed in a vacuum raised the amounts of Cr^4+^, Cr^5+^, Cr^6+^, and O_v_ and reduced the Cr^2+^ amount, all of which might aid in generating hole carriers in Cr_2_O_3_.

## Conclusions

The spin-coating process with a sol–gel precursor prepared the Cr_2_O_3_ layer. Without doping, the hole carriers of spin-coated Cr_2_O_3_ could be activated successfully by postannealing at temperatures exceeding 500 °C in a vacuum. The sheet hole concentration could reach 5 × 10^14^ cm^–2^ at a postannealing temperature of 680 °C in a vacuum. However, activating the hole carriers by annealing Cr_2_O_3_ in N_2_ ambient at a low pressure of 45 mTorr and a temperature of 560 °C or even higher was difficult. The N_2_-annealed Cr_2_O_3_ could regain the same electrical properties as vacuum-annealed Cr_2_O_3_ by being annealed in a vacuum again. A comparison of the XPS spectra of the postannealed Cr_2_O_3_ in a vacuum showed that the amounts of Cr^2+^, Cr^4+^, Cr^5+^, Cr^6+^, and O_v_ were strongly correlated with the hole carrier creation of Cr_2_O_3_.

## Supplementary Material



## Notes

Figures in Supporting Information show the measured temperature-dependent hole concentration and its fitting line of Cr_2_O_3_ annealed at 500 °C, 560 °C, and 680 °C in a vacuum, and 560 °C reannealed the N_2_-annealed Cr_2_O_3_ in a vacuum (PDF)
