# Supplementary material for: P‑Type Spin-Coated Sol–Gel Cr2O3 Layer by Postannealing in Vacuum
Source: ACS Omega. 2025 Aug 5;10(32):35763–70. doi: 10.1021/acsomega.5c02540 (PMC12368625; doi:10.1021/acsomega.5c02540)
Supplement: Supplementary file 1 [file ao5c02540_si_001.pdf]

Supporting information

P-type spin-coated sol-gel Cr_2O_3 layer by post-annealing in vacuum

Wei-Chih Lai[a, b, c,*], Chan-Hung Hsu[d], Bo-Ting Wu[a], Po-Han Chen[a], Sheng-Po Chang[e], Cheng-Huang Kuo[f], Jinn-Kong Sheu[a,c], and Shoou-Jinn Chang[g]

[a.]Department of Photonics, National Cheng Kung University, Tainan 70101, Taiwan

[b.]Research Center for Energy Technology and Strategy, National Cheng Kung University, Tainan 70101, Taiwan

[c.]Program on Key Materials, Academy of Innovative Semiconductor and Sustainable Manufacturing, National Cheng Kung University, Tainan 70101, Taiwan

[d.]Master Degree on Nano-integrated Circuit Engineering and Department of Electrical Engineering, National Cheng Kung University, Tainan 70101, Taiwan

[e.]Department of Microelectronics Engineering, National Kaohsiung University of Science and Technology, Kaohsiung 81157, Taiwan

[f.]Institute of Lighting and Energy Photonics, College of Photonics, National Yang Ming Chiao Tung University, Tainan 71150, Taiwan

[g.]Institute of Microelectronics and Department of Electrical Engineering, National Cheng Kung University, Tainan 70101, Taiwan

* Email: weilai@ncku.edu.tww

Figure S1 presents the Hall measurement of the temperature-dependent hole concentrations of Cr_2O_3 with different annealing conditions. The hole activation energies of Cr_2O_3 with different annealing conditions were extracted from fitting their temperature-dependent hole concentrations.

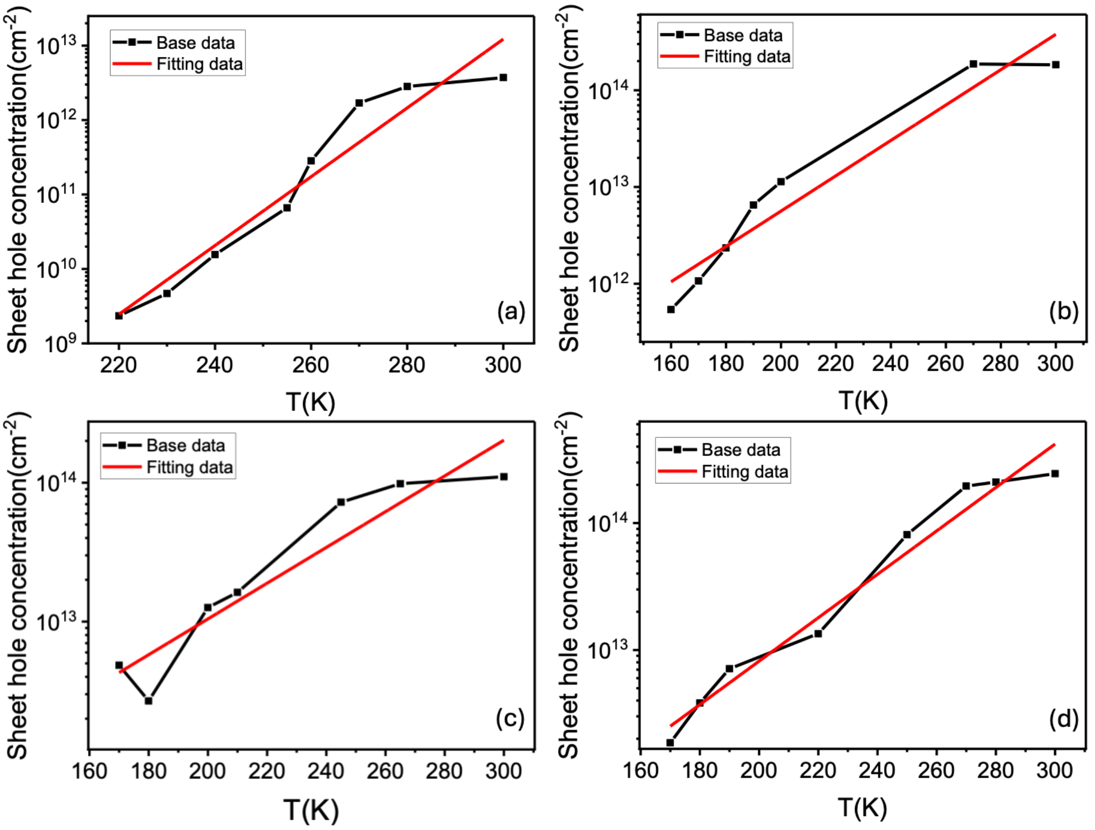

Figure S1 The measured temperature-dependent hole concentration and its fitting line of Cr_2O_3 annealed at (a) 500 °C, (b) 560 °C, and (c) 680 °C in vacuum and (d) 560 °C reannealed the N_2-annealed Cr_2O_3 in vacuum.
